# Targeted Modification of Gene Function Exploiting Homology-Directed Repair of TALEN-Mediated Double-Strand Breaks in Barley

**DOI:** 10.1534/g3.115.018762

**Published:** 2015-07-06

**Authors:** Nagaveni Budhagatapalli, Twan Rutten, Maia Gurushidze, Jochen Kumlehn, Goetz Hensel

**Affiliations:** * Plant Reproductive Biology, Leibniz Institute of Plant Genetics and Crop Plant Research (IPK), D-06466 Stadt Seeland/OT Gatersleben, Germany; † Structural Cell Biology, Leibniz Institute of Plant Genetics and Crop Plant Research (IPK), D-06466 Stadt Seeland/OT Gatersleben, Germany

**Keywords:** allele conversion, barley, designer endonuclease, targeted gene modification

## Abstract

Transcription activator-like effector nucleases open up new opportunities for targeted mutagenesis in eukaryotic genomes. Similar to zinc-finger nucleases, sequence-specific DNA-binding domains can be fused with effector domains like the nucleolytically active part of FokI to induce double-strand breaks and thereby modify the host genome on a predefined target site via nonhomologous end joining. More sophisticated applications of programmable endonucleases involve the use of a DNA repair template facilitating homology-directed repair (HDR) so as to create predefined rather than random DNA sequence modifications. The aim of this study was to demonstrate the feasibility of editing the barley genome by precisely modifying a defined target DNA sequence resulting in a predicted alteration of gene function. We used *gfp*-specific transcription activator-like effector nucleases along with a repair template that, via HDR, facilitates conversion of *gfp* into *yfp*, which is associated with a single amino acid exchange in the gene product. As a result of co-bombardment of leaf epidermis, we detected yellow fluorescent protein accumulation in about three of 100 mutated cells. The creation of a functional *yfp* gene via HDR was unambiguously confirmed by sequencing of the respective genomic site. In addition to the allele conversion accomplished *in planta*, a readily screenable marker system is introduced that might be useful for optimization approaches in the field of genome editing.

Transcription activator-like effector nucleases (TALENs) offer exciting opportunities for targeted mutagenesis. Like the zinc-finger nucleases (ZFNs), the TALENs comprise a target sequence-specific DNA-binding domain fused with the nucleolytically active part of endonuclease FokI that induces the formation of double-strand breaks (DSBs); random mutations arise at the target site as a result of error-prone DNA repair effected via nonhomologous end joining (NHEJ). First established in yeast and human cell lines, numerous other genomes are now known to be amenable to this mode of genome modification (Joung and Sander 2013). ZFNs, TALENs, and the more recently developed RNA-guided endonuclease (RGEN, also called CRISPR/Cas9) system (for review, see Voytas 2013) have yet to be widely exploited in plant genomes, but it has recently been demonstrated that heritable TALEN-mediated mutagenesis can be achieved with a relatively high efficiency in barley (Gurushidze *et al.* 2014). An attempt to use TALEN and RGEN technology to mutagenize the orthologs of the barley susceptibility factor MLO in hexaploid wheat resulted in approximately 6% of plants mutated in at least one of the three homeologous genes (Wang *et al.* 2014). An advantageous feature of the TALENs is the high modularity of the nucleotide-binding repeats, which allows for the assembly of binding domains targeting almost any site of choice in the host genome (Reyon *et al.* 2012; Schmidt-Burgk *et al.* 2013). Because FokI activity is only manifested as a dimer, two TALEN units need to be expressed simultaneously. Consequently, the target site comprises about 50 nt, sufficient to ensure a high degree of specificity.

The inclusion of a DNA repair template facilitates homology-directed repair (HDR), which has been shown to increase the chances of effecting predefined rather than random DNA sequence modifications. The NHEJ repair frequency of DSBs induced within a reporter gene, as generated by ectopically expressing I-*Sce*I endonuclease in barley, lay in the range 47–58%, including some 1−3 nt indels (Vu *et al.* 2014). A further 1–8% of the modifications displayed synthesis-dependent strand annealing associated with NHEJ, whereas 20–33% arose from homologous recombination. The surprisingly high proportion of HDR has encouraged the possibility of deploying a repair template-mediated genome editing approach.

In plants, homologous recombination-based modifications have been largely limited to alterations resulting in a selectable or a screenable phenotype (reviewed by Voytas 2013). Recent examples of sequence knock-in (targeted insertion) employing RGEN-mediated DSB induction have been reported in both rice and *Nicotiana benthamiana* protoplasts (Li *et al.* 2013; Shan *et al.* 2013), in which the templates were between 72 and 647 bp in length; the resulting rate of incorporation of restriction enzyme recognition sites into the target loci was 6–10%. More recently, Schiml *et al.* (2014) achieved heritable genome modifications by integrating a resistance gene cassette into an endogenous *ADH1* locus via *in planta* gene targeting after RGEN-mediated DSB induction. In this context, these authors suggested that the directed exchange of single base pairs within the coding sequence of a protein of interest is one of the challenges still needed to be addressed using *in planta* gene-targeting technology. Baltes *et al. *(2014), working with *Nicotiana tabacum*, used circular geminivirus replicons to introduce sequence-specific nucleases as well as DNA repair template, and showed that ZFN, TALEN, and RGEN elements could all be expressed and then induce mutation at a high frequency. A nonfunctional *GUS:NPTII* transgene was corrected by transforming tobacco leaves with these geminivirus replicons, reconstituting a functional *GUS* copy. However, so far, repair template-mediated conversion of a functional allele toward the gain of altered gene functionality has not yet been reported for any plant host.

Here we exploit TALEN technology involving HDR of DSBs to generate a precisely directed genome modification at the individual nucleotide level *in planta*. This approach represents a novelty in monocotyledons, which are of utmost agricultural importance. To exemplify the principle, a stably integrated *gfp* gene is converted into *yfp*, which is readily screenable at the cellular level. The two gene products differ only in a single T203Y amino acid, which causes an altered light emission spectrum (Wachter *et al.* 1998). We show that yellow fluorescing cells can be reproducibly generated via allele conversion and unambiguously confirm the genetic modification by sequencing the genomic target site.

## Materials and Methods

### Plant materials

**Figure 1:**
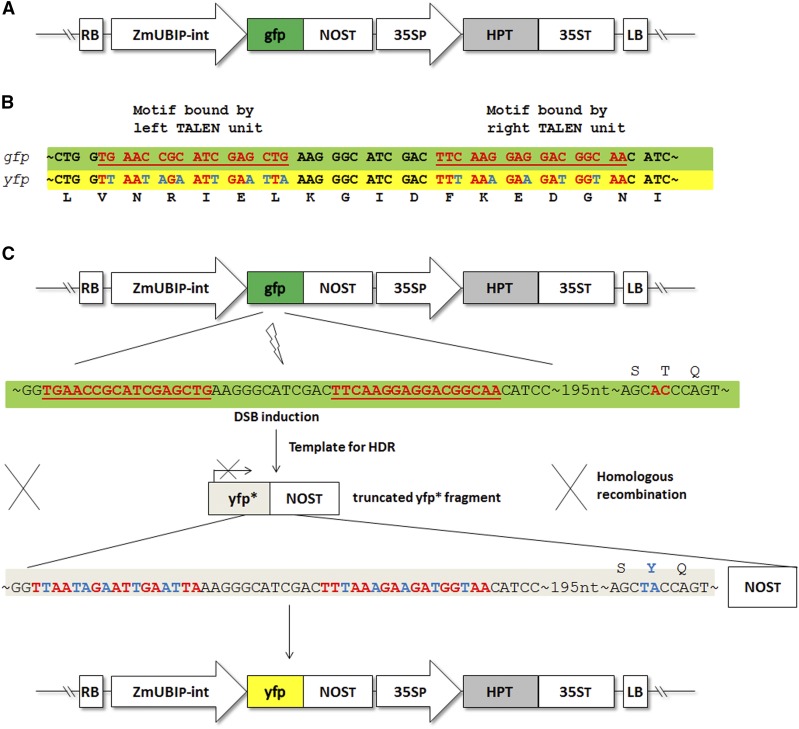
Concept of homology-directed repair (HDR) of transcription activator-like effector nuclease (TALEN)-induced double-stand breaks (DSBs) in barley. (A) Barley genomic region with transfer DNA (T-DNA) of *gfp* plants used for retransformation with *gfp*-specific TALEN constructs. (B) *gfp*-specific TALEN target site (with DNA-motifs bound by the left and right TALEN units indicated in red and underlined) aligned with the respective sequence of the truncated *yfp* fragment (yfp*) used as a template for HDR, which is modified by substitution with synonymous codons (in blue) to prevent the TALENs from binding, while the fluorescent protein (XFP) amino acid sequence is retained. (C) Mechanism showing the *gfp* TALEN-induced DSBs in T-DNA region of *gfp* and used truncated yfp* fragment as template for HDR, leading to conversion of *gfp* to *yfp*.

The two transgenic winter barley (*Hordeum vulgare*) cv. ‘Igri’ lines, denoted BPI 09 and BPI 11, each carry a single copy of *gfp* (Figure 1A). The grain was germinated (14/12° day/night, 16-hr photoperiod at 20,000 lux), and the seedlings vernalized for 8 wk (4°, 9-hr photoperiod), then raised in a glasshouse (18/14°, 16-hr photoperiod at ∼25,000 lux). Spikes were harvested when the awns had just emerged from the flag leaf, as described by Kumlehn *et al.* (2006).

### Plasmids and transgenic barley plants

A pair of *gfp*-specific TALEN constructs pGH297 (carrying the left-hand unit) and pGH400 (right-hand unit) was generated as described by Gurushidze *et al.* (2014) (Supporting Information, Figure S1, A and B). A fragment comprising *yfp* gene along with *NOPALINE SYNTHASE* terminator were synthesized and introduced into *Eco*RV cloning site in pUC57 at GenScript (Piscataway, NJ), then subsequently cloned at *Bst*XI/*Bam*HI sites into pYF133 (Fang *et al.* 2002) [pGFP in this study, Figure S1C] to generate pGH418, in which *gfp* was replaced by *yfp* [pYFP, in this study, Figure S1D]. To avoid the possibility of the *gfp*-TALEN−induced mutation of *yfp*, its binding sites were modified by introducing synonymous codons, leaving the polypeptide sequence unaltered (Figure 1B). Electroporation was used to introduce the TALEN constructs into *Agrobacterium tumefaciens* strain LBA4404/pSB1, which harbors the disarmed Ti plasmid pAL4404 and the hypervirulence-conferring plasmid pSB1 (Komari *et al.* 1996). Microspore isolation and the agroinoculation of embryogenic BPI 09 and BPI 11 pollen with each of the two *gfp*-specific TALEN constructs were performed following Kumlehn *et al.* (2006), except that the population density of the transformed LBA4404/pSB1 cells was adjusted to 2.5 × 10^7^ colony-forming units per milliliter. Bialaphos was used as the selective agent. The regenerants were screened for the presence and expression of *Fok*I nuclease and two lines showing the expression of the right- (335R and 338R) and another two lines of the left-hand (319L and 462L) *gfp*-TALEN units were carried forward.

### Biolistic transformation of leaf cells

Gold particles of diameter 1.0 µm (Bio-Rad, Munich, Germany) were coated with DNA, and a 1.5-mg aliquot used for each shot. The concentration of coating DNA was 1 µg/µL (pGH297, pGH400) and 2−3 µg/µL (834 bp *Btg*ZI/*Bss*HII-linearized and gel-purified pUC57-yfp DNA fragment), precipitated from solution according to Sanford *et al.* (1991). The transformation was effected using a Biolistic Particle Delivery System-1000/He device equipped with 1100 psi rupture disks (Bio-Rad, Munich, Germany). Five primary leaves sampled from 7- to 10-d-old seedlings were laid adaxial side up on 1% agar containing 20 µg/mL benzimidazol and 20 µg/mL chloramphenicol. After bombardment, the leaf material was held at room temperature for approximately 24 hr before being subjected to fluorescence microscopy.

### Biolistic transformation of embryo cells

Immature grains (12−16 d after anthesis) were surface sterilized by immersion for 3 min in 70% (v/v) ethanol, followed by 20 min in 2.4% (w/v) sodium hypochlorite plus 0.1% (v/v) Tween 20 and four rinses in sterile distilled water. The embryos were dissected out aseptically and cultured for 5 d at 24° in the dark on a solid (3 g/L phytagel) medium containing 4.4 g/L Murashige and Skoog salts, 112 g/L B5 vitamins, 30 g/L sucrose, 5 μM copper sulfate, and 5 mg/L dicamba (all chemicals were purchased from Duchefa, Haarlem, The Netherlands). Before bombardment, the callus was transferred onto the same medium supplemented with 0.4 M sorbitol for 4 hr. A 3-mg aliquot of DNA-coated gold particles, prepared as described in the previous paragraph, was used to bombard 20 calli, employing the same device as listed previously, but using a 900 rather than a 1100 psi rupture disk. After bombardment, the material was held for approximately 24 hr at 24° in the dark before being subjected to fluorescence microscopy.

### Confocal microscopy

The DSB induction efficiency caused by the TALEN constructs and that of their subsequent HDR was determined by establishing the ratio of *yfp*-expressing and *gfp*-nonexpressing cells in five 2 × 1-cm leaf segments. Only cells from the stomatal cell rows (guard cells and adjacent epidermal cells, see Figure S2) were counted. GFP (488 nm excitation, 491−530 nm emission) and YFP (514 nm excitation, 517−560 nm emission) signals were captured by confocal microscopy, and the identity of the fluorophores was confirmed by photospectrometric analysis.

### Genomic DNA isolation and polymerase chain reaction (PCR)

Genomic DNA was isolated from snap-frozen transiently transformed leaves following the protocol of Palotta *et al.* (2000). Each subsequent 20 μL of PCR contained 50−100 ng template and was based on the primer pairs listed in Table S1 and standard PCR conditions. The reaction products were purified using a QIAquick PCR Purification kit (QIAGEN, Hilden, Germany) for subsequent sequencing.

### RNA isolation and reverse transcription

We isolated total RNA by using the TRIzol reagent (Life Technologies, Darmstadt, Germany), following the manufacturer’s protocol. After DNase I treatment (DNA-free kit, Life Technologies), the first cDNA strand was synthesized using an iScript Select cDNA synthesis kit (Bio-Rad). The resulting cDNA served as the template for subsequent reverse-transcription PCRs based on the *Fok*I domain primer pair (Figure S1E).

### Data availability

Plasmids, strains and grains of transgenic barley lines are available upon request.

## Results and Discussion

Our aim here was to demonstrate the feasibility of editing a plant genome by precisely modifying a defined target DNA motif. A *gfp*-specific TALEN was used in conjunction with a repair template which, via HDR, was intended to convert *gfp* into *yfp*, an alteration achievable by inducing a T203Y substitution (Figure 1C). Such an alteration would be readily detectable, as it produces a shift in fluorescence emission from 509 nm (GFP) to 527 nm (YFP) (Wachter *et al.* 1998).

### TALEN-mediated allele conversion in barley immature embryos

In an initial experiment, immature embryos of barley transgenic lines harboring *gfp* (Figure 1A) and actively expressing either (1) the left-hand *gfp*-TALEN unit (line 462L) or (2) the right-hand *gfp*-TALEN unit (line 335R) were co-bombarded with vectors carrying the complementary *gfp*-TALEN unit (pGH400 and pGH297, respectively, Figure S1, A and B) and a linearized, truncated, nonfunctional yfp* fragment (Figure 1C). To confirm proper expression of the integrated TALEN units, semiquantitative reverse-transcription-PCR using a primer pair specific for *Fok*I was performed (Figure S1E). The design of the *gfp*-TALEN pair and the generation of transgenic plants essentially followed Gurushidze *et al.* (2014). To prevent TALEN-mediated mutations in the repair template, its TALEN recognition sequences were modified by substitution with synonymous codons, which retained the YFP amino acid sequence (Figure 1B). One day after bombardment a number of yellow fluorescing cells were detected (Figure S3, A and E) and confirmed by corresponding emission peaks recorded by the confocal laser scanning microscope (Figure S3B). Both GFP and YFP fluorescence were clearly detectable, indicating high rates of gene expression. The turnover rate, in contrast, also may be significant, considering that some of the cells apparently lacked GFP fluorescence while showing clear YFP activity (Figure S3, C−E).

### Allele conversion frequently occurs in leaves

To assess the frequency of allele conversion, leaves of the homozygous *gfp* barley plants additionally expressing either the left-hand (lines 319L and 462L) or the right-hand (lines 335R and 338R) members of the *gfp*-TALEN pair were bombarded as described previously. After bombardment, 2-cm long leaf segments were analyzed with regard to the proportion of the total number of cells (stomatal complex and the first layer of adjacent epidermal cells), the nonfluorescent cells, and the ones showing yellow fluorescence (Figure S2). Possible scenarios in treated cells with regards to their fluorescence are detailed in Table 1.

**Table 1:** Possible scenario after bombardment of leaf material

Scenario	Fluorescence
(G1) nontransformed cells (not met by a DNA-coated gold particle)	Green
(G2) transformed cells where TALENs did not create a DSB in the target site	
(G3) cells with both *gfp* alleles being unaltered owing to correct repair or with a mutation not affecting gene function	
(G4) cells with one *gfp* allele being functionally affected via NHEJ, whereas the other one is unaltered or its function is retained despite of a (minor) mutation	
(GY1) cells with one *gfp* allele being modified by HDR using the yfp*-repair template, whereas the other one remained functionally unaffected	Green and yellow
(Y1) cells with both *gfp* alleles being modified by HDR using the yfp*-repair template	Yellow
(Y2) cells with one *gfp* allele being modified by HDR using the yfp*-repair template, whereas the other one is functionally affected by NHEJ	
(N1) cells with both *gfp* alleles being functionally affected by NHEJ	No
(N2) cells that have not survived	

TALENs, transcription activator-like effector nucleases; DSB, double-strand break; NHEJ, nonhomologous end joining; HDR, homology-directed repair; yfp*, truncated yellow fluorescent protein.

**Figure 2:**
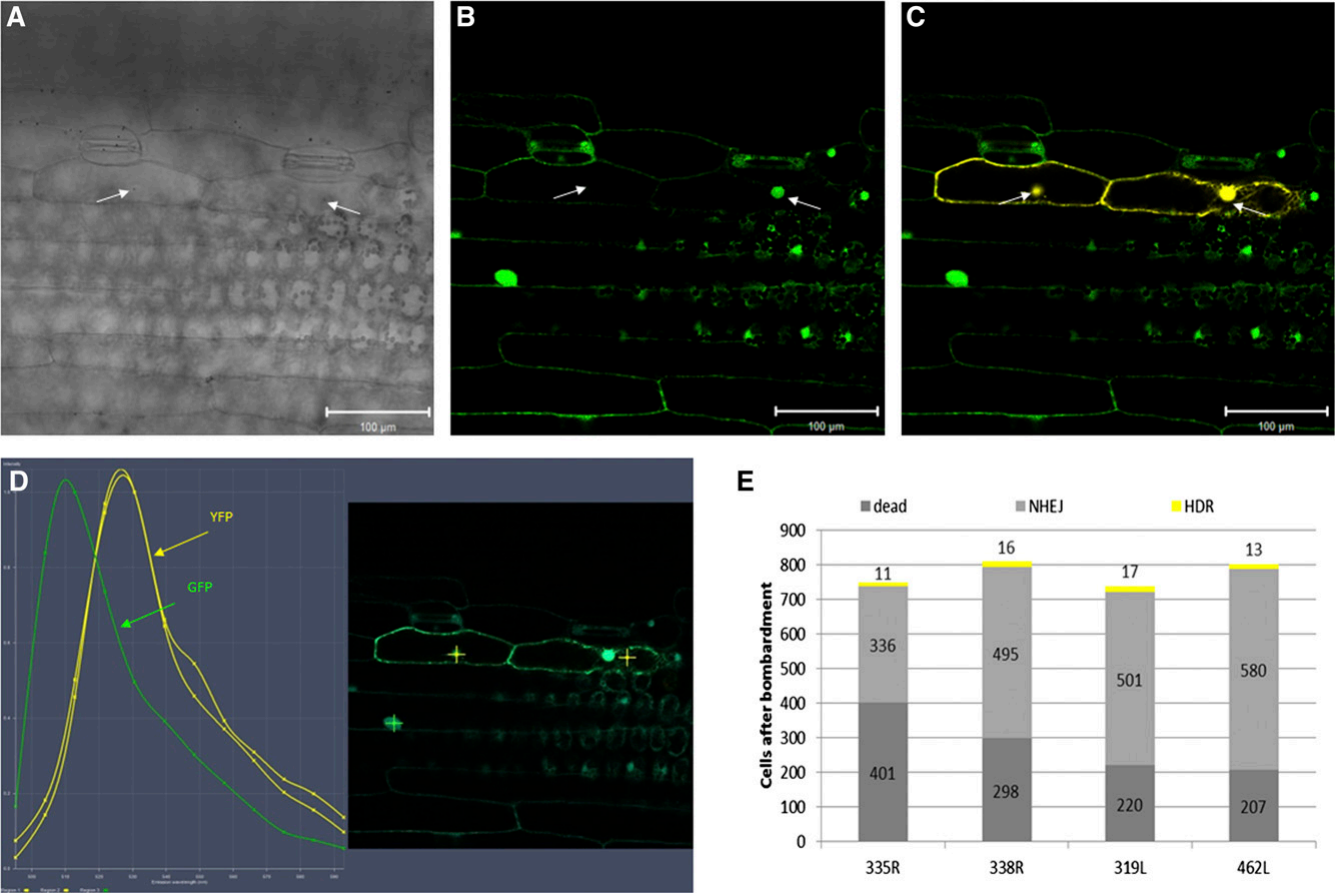
Homology-directed repair (HDR) after the induction of TALEN-mediated double-stand breaks (DSBs) in barley. (A) Bright field of a leaf of line 319L (carrying *gfp* and the left-hand TALEN unit) taken 24 hr after bombardment with the right-hand TALEN unit and linearized yfp* fragment. (B) Epifluorescence of the same leaf shown in (A) after excitation with 488-nm laser light and the spectral unmixing of green fluorescent protein (GFP) using confocal microscopy. (C) Epifluorescence of the same leaf shown in (A) after excitation with 488-nm laser light and the spectral unmixing of GFP (green) and yellow fluorescent protein (YFP; yellow) signals using confocal microscopy. Bright field, GFP and merged GFP and YFP signals. Arrows indicate nuclei of two adjacent epidermal cells. Bar: 100 µm and (D) Lambda stack of same materials shown in (A−C) used to visualize the presence of GFP (emission peak at 509 nm) and YFP (527 nm). (E) The bombardment of lines 319L and 462L (carrying *gfp* and the left-hand TALEN unit), and 335R and 338R (*gfp* plus the right-hand TALEN unit). Bars represent the number of nonfluorescing cells resulting either from mechanical damage (dark gray, numbers inferred from control bombardments in the absence of the complementary TALEN unit) or from TALEN-mediated DSB followed by error-prone NHEJ (light gray). Events resulting from TALEN-mediated DSB followed by HDR in the presence of the yfp* repair template are shown in yellow. Cells expressing *gfp* only are not indicated. Each experiment was conducted on two independent transgenic plant lines. Bars represent the mean of five leaves (see Table S2).

After bombardment of negative control samples using the very same *gfp*-TALEN unit as was already stably integrated in the plant material used (*e.g.*, bombardment of line 338R using pGH400 also carrying the right TALEN unit), ca. 1% (207 of 20,238 cells) of the cells failed to show green fluorescence, suggesting these to represent the rate of cell death due to mechanical damage (Figure 2E, dark gray bars). A further ca. 2% (495 of 19,141 cells) could be accounted for by random mutagenesis at the TALEN target site when complementary *gfp*-TALEN units were used without the yfp* repair template (Figure 2E, light gray bars) (*e.g.*, bombardment of line 319L with pGH400 carrying the right TALEN unit). YFP fluorescence, confirmed by corresponding emission peaks recorded by the confocal laser scanning microscope (Figure 2D), was only observed when the treatment involved the truncated yfp* repair template and both complementary *gfp*-TALEN units (Table S2 and Figure 2, A−C). Under this condition, on average 2–3% of the cells mutated at the target site were found to express *yfp*, thereby displaying a conversion of at least one *gfp* allele into *yfp*, which is a reasonable success rate of precise genome editing (Table S2).

In the present study, the first example is provided for plants, where a functional allele gains modified functionality after repair of TALEN-mediated DSBs. Townsend *et al.* (2009) have previously demonstrated by the ZFN approach using tobacco protoplasts that the introduction of point mutations in *SurA* or *SurB* causes a loss of binding capacity between the concerned gene products and the herbicides chlorsulfurone and imazaquin, which is associated with an advantage under selective conditions. More recently, the same approach was conducted using TALENs instead of ZFNs which resulted in an efficiency of 4% of allele replacement (Zhang *et al.* 2013). In maize it was shown that simultaneous expression of ZFNs and delivery of a simple heterologous donor molecule leads to precise targeted addition of an herbicide-tolerance gene at the intended locus in a significant number of isolated events (Shukla *et al.* 2009). Other previous examples of functional restoration of target genes rested on RGEN-mediated DSB induction without use of repair template (Feng *et al.* 2013; Jiang *et al.* 2013; Baltes *et al.* 2014) or on endonuclease I-*Sce*I, the applicability of which is largely confined to its native DNA target sequence (Fauser *et al.* 2014).

### Genomic integration of the allele conversion was confirmed by sequencing

**Figure 3:**
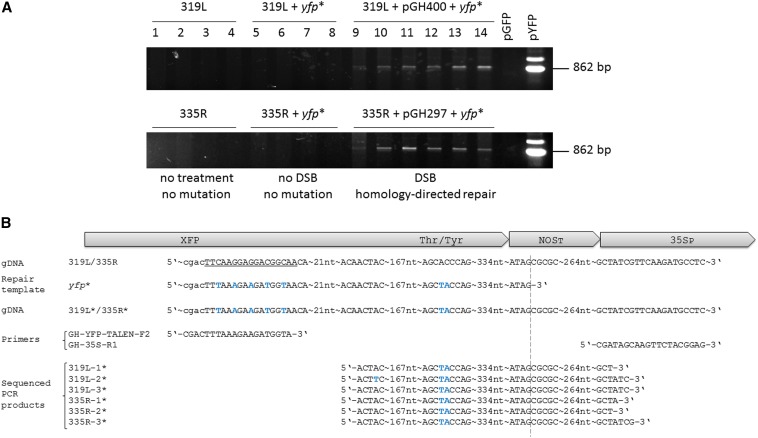
Polymerase chain reaction (PCR) analysis and DNA-sequencing confirm *gfp*-to-*yfp* conversion within the host genome. (A) PCR products amplified from genomic DNA extracted from leaves of lines 319L (upper gel) and 335R (lower gel) without bombardment are shown in lanes 1−4, of those bombarded with the linearized truncated yfp* fragment alone in lanes 5−8, and of those bombarded with both the linearized truncated yfp* fragment and the complementary TALEN unit (pGH400 for right-hand, pGH297 for the left-hand unit) in lanes 9−14. pGFP was the negative control [plasmid carrying *gfp* transfer DNA (T-DNA)] and pYFP positive control (plasmid carrying *yfp* T-DNA). (B) Verification of allele conversion via sequencing; presented are sequences of donor plant (gDNA), the truncated yfp* fragment used as repair template, the genomic target sequence after homology-directed repair of TALEN-mediated DSBs, forward and reverse primers used for DNA-amplification from samples of different leaves of lines 319L (left-hand TALEN unit) and 335R (right-hand TALEN unit) 24 hr after bombardment as well as sequencing data providing evidence of *gfp*-to-*yfp* conversion. The sequences recovered from all other independent leaves were identical to those three per plant line shown here. Nucleotides different in the yfp* sequence from those of *gfp* are printed in blue. The binding site of the right TALEN unit is underlined. An asterisk indicates an altered target sequence after leaf bombardment in lines 339L and 335R. The dotted line indicates the end of the repair template. XFP, green or yellow fluorescent protein; Thr/Tyr, amino acid change responsible for GFP-to-YFP conversion; NOST, *A. tumefaciens NOPALINE SYNTHASE* transcriptional termination sequence; 35SP, CaMV *35S* promoter.

Because somatic cells are diploid and therefore carry two copies of each gene, the loss of GFP activity would require the loss-of-function of both copies. Therefore, mutation efficiency is easily underestimated. A number of the *yfp*-expressing cells coexpressed *gfp*, but others clearly showed solely YFP activity (Figure 2, A−C, left cell, Figure S3, C-E). This fact emphasizes how fast the process of DNA repair after TALEN-mediated DSB induction can take place. In contrast, it was somehow surprising that in yellow fluorescent cells 1 d after bombardment, the GFP protein was already depleted to below the detection limit. To confirm that the genomic *gfp* sequence had been converted into the *yfp*, PCR was conducted using primers (Table S1) specific for *yfp* and the *35S* promoter driving the selectable marker gene (*HPT*), the latter expression unit being present within the stably inserted transfer DNA that also carries the *gfp* gene. Thus, an amplicon could only be generated if a TALEN-induced DSB had been processed by HDR, based on the *yfp* fragment supplied in the form of the repair template (Figure 3A). Sequencing of the fragments recovered from the amplicons unambiguously confirmed that the desired conversion had occurred in the genomic DNA (Figure 3B). The principle of *gfp*-to-*yfp* conversion introduced in this study might be instrumental in future studies on genome editing. For instance, one could optimize various properties of customizable endonucleases like TALEN spacer length, target motif composition, as well as editing-related features such as the size of the repair template, length of homologous regions, or overexpression of genes involved in the HDR process. A challenge that remains to be overcome before the principle demonstrated here at the cellular level can be extended to the regeneration of plants carrying a precisely predicted modification is that such genome editing events are not typically associated with any selective advantage. As a result, alternatives to the conventional use of selective agents (such as herbicide or antibiotic resistance) will need to be elaborated. However, for the moment, what has been demonstrated is the proof of concept that any plant gene can be modified in a predictable way, thereby opening the door to redesigning native gene sequences to modify their function or to reverse mutations in a directed way.

Predictable genetic modifications comprising only a few genomic base pairs rather than entire genes are of particular practical relevance, because they might not fall under the European regulation of genetically engineered organisms. One prominent example where the precise editing of only a small genomic motif may result in a valuable trait is the wheat Lr34 ABC transporter, which confers durable resistance to multiple fungal pathogens (Krattinger *et al.* 2009). The authors showed that resistance was associated with either the deletion of a phenylalanine residue (M19) or a single-nucleotide polymorphism in exon 12 which causes a tyrosine to a histidine (M21) conversion. Likewise, small alterations in the nucleotide sequence associated with valuable gain or modification of function have been shown in many other plant genes, *e.g.*, those involved in resistance or flowering time (Ruben *et al.* 2006; Dally *et al.* 2014). The capacity to achieve this would represent a quantum improvement over established methods of random mutagenesis or the knock-in of an entire coding sequence. The technique promises to revolutionize the way plant genes are functionally validated, not to mention to create opportunities for crop improvement based on the paradigm that any sequence-defined allelic variant can be established at its native locus without perturbing the genetic background.

TALEN-mediated targeted mutation has been shown previously in a variety of plant species. The results presented here provide evidence that targeted allele conversion predictably gaining a new function is also feasible. Although previous reports showing that customized repair templates can be used for HDR of DSBs in plants have been largely confined to dicots like Arabidopsis or tobacco, a first example of this approach is given here using a Triticeae tribe member with high agronomic importance. In addition, a readily screenable marker system is introduced that might be useful for methodological optimization studies in the field of genome editing.

## Supplementary Material

Supporting Information
