# Supplementary material for: Targeted Modification of Gene Function Exploiting Homology-Directed Repair of TALEN-Mediated Double-Strand Breaks in Barley
Source: G3 (Bethesda). 2015 Jul 6;5(9):1857–63. doi: 10.1534/g3.115.018762 (PMC4555222; doi:10.1534/g3.115.018762)
Supplement: Supporting Information [file supp_5_9_1857__index.html]

Targeted Modification of Gene Function Exploiting Homology-Directed Repair of TALEN-Mediated Double-Strand Breaks in Barley — Supporting Information 

# Targeted Modification of Gene Function Exploiting Homology-Directed Repair of TALEN-Mediated Double-Strand Breaks in Barley

## Supporting Information for Budhagatapalli *et al.*, 2015

**Files in this Data Supplement:**

- Supporting Information - Figures S1-S3 and Tables S1-S2 (PDF, 2 MB)
- Figure S1 - Details of the binary plasmids used in the study and expression data of *Fok*I in the leaves of donor material. (PDF, 1014 KB)
- Figure S2 - Confocal microscopy image of the barley abaxial leaf surface demonstrating the cell types present. (PDF, 362 KB)
- Figure S3 - HDR following the induction of TALEN-mediated DSBs in cultured immature barley embryos. (PDF, 773 KB)
- Table S1 - List of primers used for the identification of T-DNA elements in the study. (PDF, 293 KB)
- Table S2 - Quantification of homology‐directed repair in barley leaves. (.xlsx, 13 KB)
