# Supplementary material for: Targeted Modification of Gene Function Exploiting Homology-Directed Repair of TALEN-Mediated Double-Strand Breaks in Barley
Source: G3 (Bethesda). 2015 Jul 6;5(9):1857–63. doi: 10.1534/g3.115.018762 (PMC4555222; doi:10.1534/g3.115.018762)
Supplement: Supporting Information [file supp_g3.115.018762_018762SI.pdf]

G3: Genes | Genomes | Genetics

Targeted modification of gene function exploiting homology-directed repair of TALEN-mediated double strand breaks in barley

Nagaveni Budhagatapalli[a], Twan Rutten[b], Maia Gurushidze[a], Jochen Kumlehn[a], and Goetz Hensel[a,1]

[a] Plant Reproductive Biology, Leibniz Institute of Plant Genetics and Crop Plant Research (IPK), Corrensstr. 3, D-06466 Stadt Seeland/OT Gatersleben, Germany
[b] Structural Cell Biology, Leibniz Institute of Plant Genetics and Crop Plant Research (IPK), Corrensstr. 3, D-06466 Stadt Seeland/OT Gatersleben, Germany

[1] To whom correspondence should be addressed.
Dr. Goetz Hensel
Plant Reproductive Biology
Leibniz Institute of Plant Genetics and Crop Plant Research (IPK)
Corrensstr. 3
D-06466 Stadt Seeland/OT Gatersleben
Germany

E-Mail: hensel@ipk-gatersleben.de
Tel: +49(0)39482-5543
Fax: +49(0)39482-5515

DOI: 10.1534/g3.115.018762

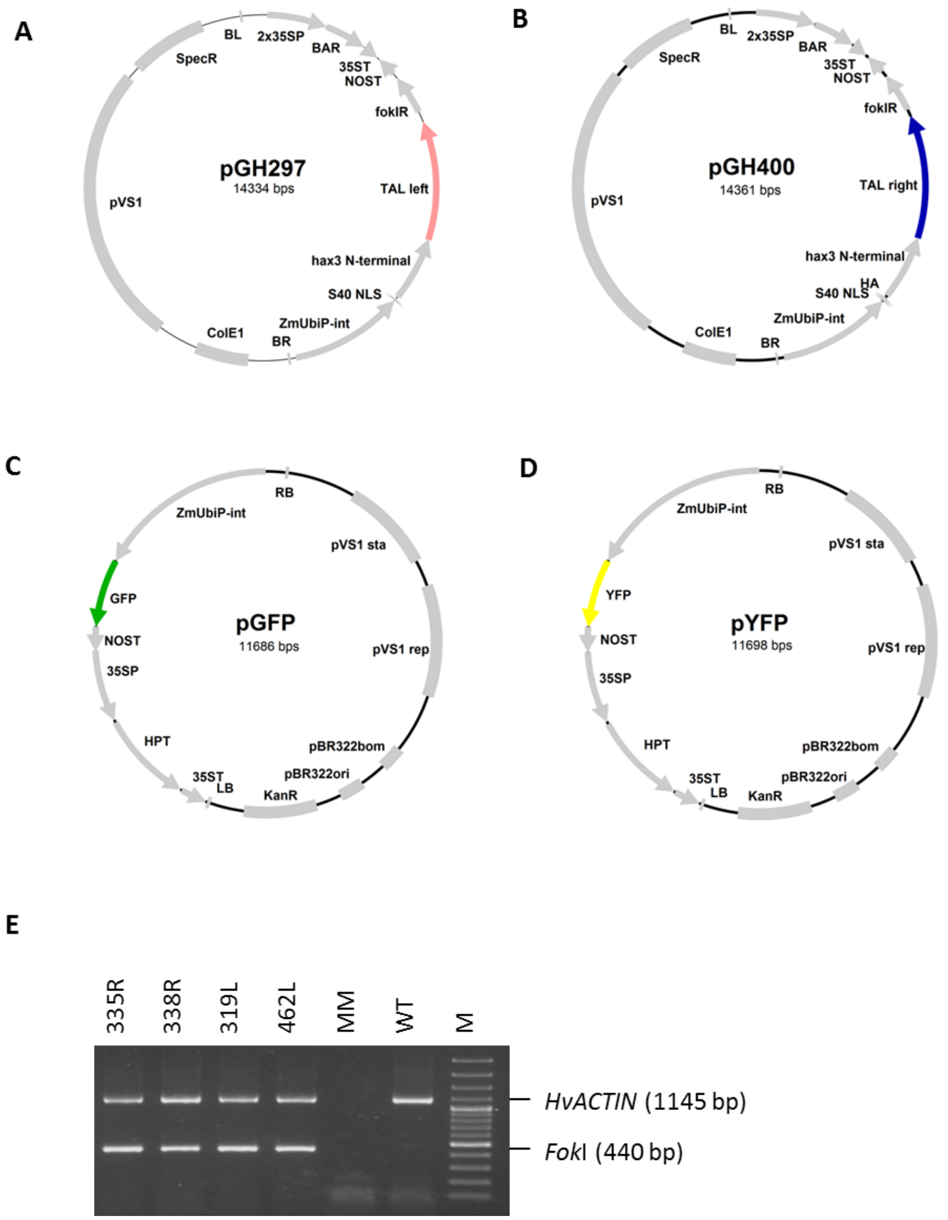

Figure S1. Details of the binary plasmids used in the study and expression data of *Fok*I in the leaves of donor material. (A) and (B) Binary plasmids used to obtain expression of the *gfp*-specific TALEN units. SpecR: *ADENYLTRANSFERASE* (encodes resistance to spectinomycin), LB: T-DNA left border, d35SP: CaMV *35S* promoter, BAR: *BAR* (encodes resistance to bialaphos), 35ST: CaMV *35S* transcriptional termination sequence, NOST: *A. tumefaciens NOPALINE SYNTHASE* transcriptional termination sequence, FokI: FokI cleavage domain, TALEN (left or right): customized binding domain of the *gfp*-TALEN units, HA: haemagglutinin tag, S40 NLS: SV40 nuclear localization signal, UBIP: maize *UBIQUITIN-1* promoter plus first intron, RB: T-DNA right border, ColE1: *E. coli* plasmid ColE1 high-copy replication origin, pVS1: *Pseudomonas aeruginosa* plasmid pVS1 replication origin. (C) Binary plasmid used for developing stable *gfp* lines and (D) binary plasmid carrying *yfp* gene cassette. (E) Transcription analysis in the presence of the *gfp*-TALEN units. The cDNAs were prepared from lines 335R and 338R (harboring the right-hand unit), and from lines 319L and 462L (left-hand unit). The *Fok*I cleavage domain was amplified using primers given in Supplemental Table S1 and *HvACTIN1* was used as the reference sequence.

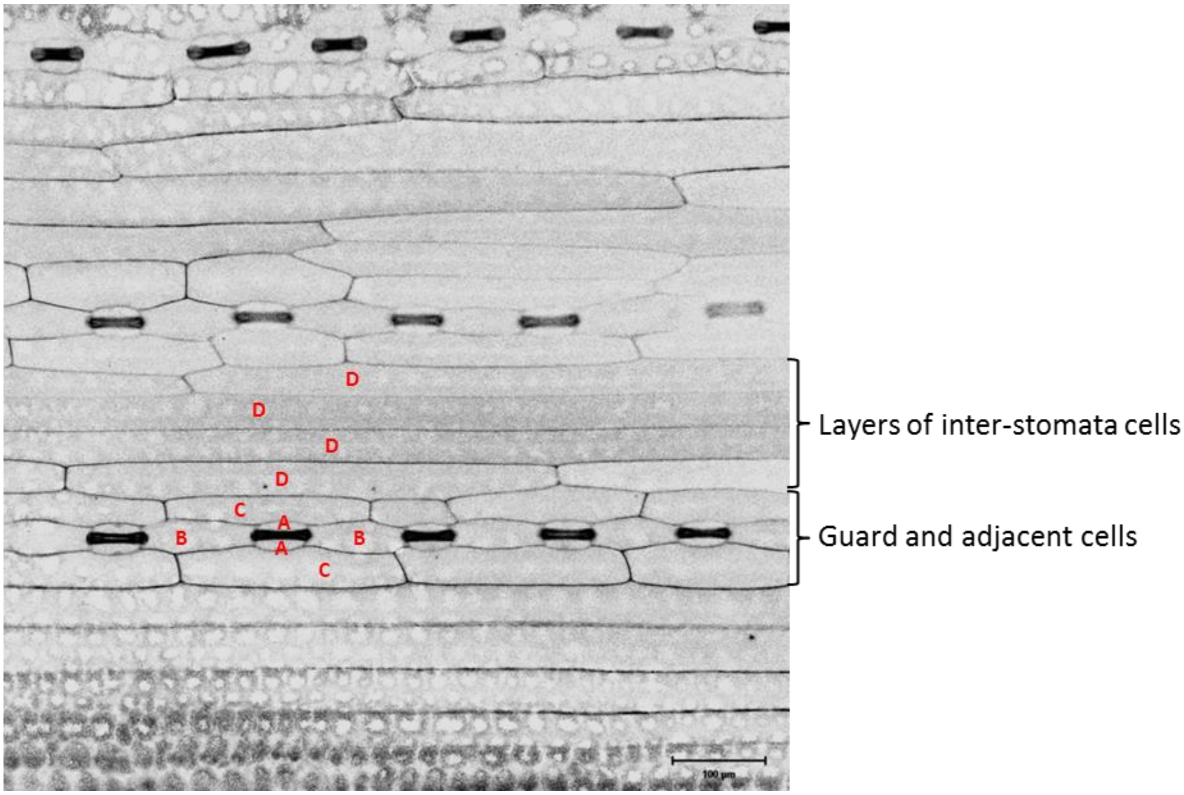

Figure S2. Confocal microscopy image of the barley abaxial leaf surface demonstrating the cell types present. The guard and adjacent epidermal cells labeled "A", "B" and "C" were included in the count, whereas those marked "D" (narrow cells in the inter-stomatal region) were excluded due to the low efficiency of transient transgene expression.

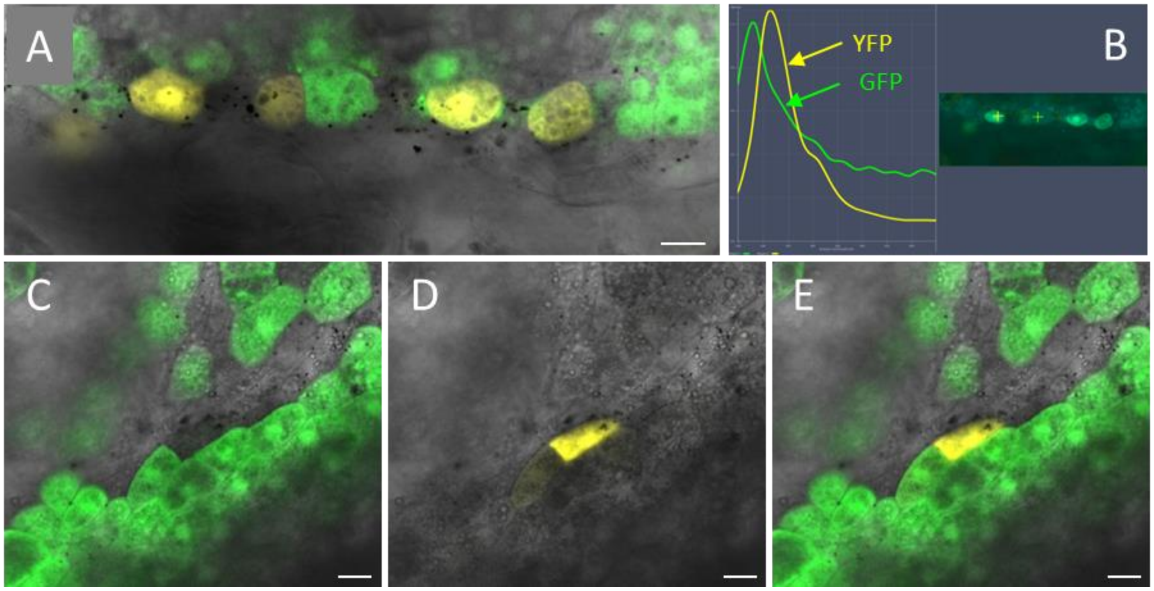

Figure S3. HDR following the induction of TALEN-mediated DSBs in cultured immature barley embryos. (A) Merged bright field and epifluorescence images of line 462L (carrying *gfp* and the left-hand TALEN unit) callus taken 24 h after bombardment with the right-hand *gfp*-TALEN unit and linearized *yfp** fragment. Bar: 20 µm. (B) Lambda stack of same materials shown in (A) used to visualize the presence of GFP (emission peak at 509 nm) and YFP (527 nm). (C; D) Epifluorescence of transiently transformed 462L callus after excitation with 488 nm laser light and spectral unmixing to identify (C) GFP and (D) YFP signals. Bar: 20 µm. (E) Merged image of (C) and (D).

Table S1. List of primers used for the identification of T-DNA elements in the study.

Primer	Sequence 5' – 3'	Amplified region, primer orientation
Actin-F1	GGATCCGATGGCTGACGGTGAGGACATCCAG	*HvACTIN1*, forward
Actin-F2	CCATGGAGAAGCACTTCCTGTGGACGATCG	*HvACTN1*, reverse
FokI-F1	ATCGAGATCGCCCGGAACAGCACC	*Fok*I gene, forward
FokI-R	ATCATCTCGCCGCCGATCAGGAGC	*Fok*I gene, reverse
GH-35S-R1	GAGGCATCTTGAACGATAGC	*CaMV 35S* promoter, reverse
GH-YFP-TALEN-F2	CGACTTTAAAGAAGATGGTA	*yfp* TALEN-right unit, forward

Table S2 Quantification of homology-directed repair in barley leaves.

Available for download as an Excel file at www.g3journal.org/lookup/suppl/doi:10.1534/g3.115.018762/-/DC1
