# Supplementary material for: Targeted Modification of Gene Function Exploiting Homology-Directed Repair of TALEN-Mediated Double-Strand Breaks in Barley
Source: G3 (Bethesda). 2015 Jul 6;5(9):1857–63. doi: 10.1534/g3.115.018762 (PMC4555222; doi:10.1534/g3.115.018762)
Supplement: Supporting Information [file supp_g3.115.018762_FigureS1.pdf]

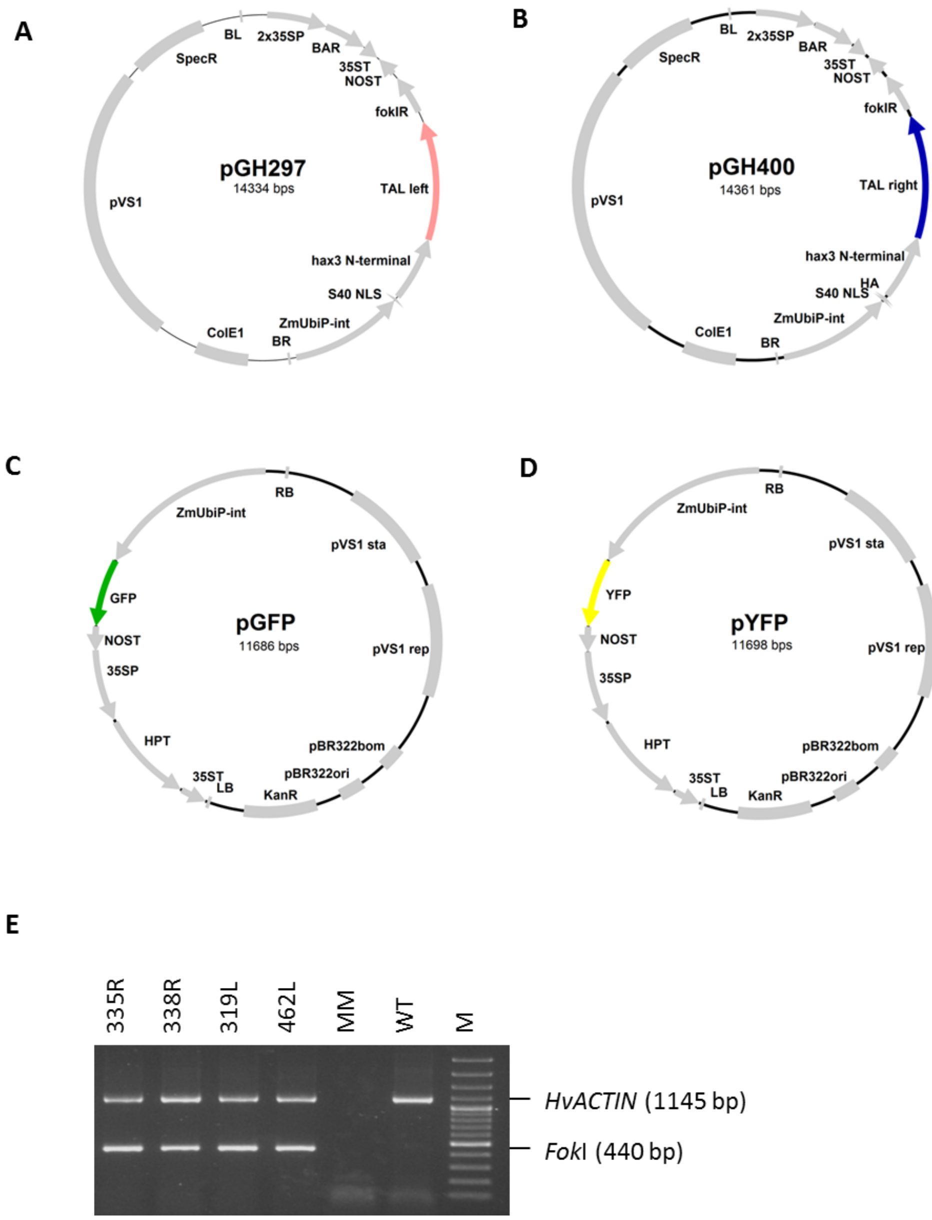

Figure S1. Details of the binary plasmids used in the study and expression data of *Fok*I in the leaves of donor material. (A) and (B) Binary plasmids used to obtain expression of the *gfp*-specific TALEN units. SpecR: *ADENYLTRANSFERASE* (encodes resistance to spectinomycin), LB: T-DNA left border, d35SP: CaMV *35S* promoter, BAR: *BAR* (encodes resistance to bialaphos), 35ST: CaMV *35S* transcriptional termination sequence, NOST: *A. tumefaciens NOPALINE SYNTHASE* transcriptional termination sequence, FokI: FokI cleavage domain, TALEN (left or right): customized binding domain of the *gfp*-TALEN units, HA: haemagglutinin tag, S40 NLS: SV40 nuclear localization signal, UBIP: maize *UBIQUITIN-1* promoter plus first intron, RB: T-DNA right border, ColE1: *E. coli* plasmid ColE1 high-copy replication origin, pVS1: *Pseudomonas aeruginosa* plasmid pVS1 replication origin. (C) Binary plasmid used for developing stable *gfp* lines and (D) binary plasmid carrying *yfp* gene cassette. (E) Transcription analysis in the presence of the *gfp*-TALEN units. The cDNAs were prepared from lines 335R and 338R (harboring the right-hand unit), and from lines 319L and 462L (left-hand unit). The *Fok*I cleavage domain was amplified using primers given in Supplemental Table S1 and *HvACTIN1* was used as the reference sequence.
