# Supplementary material for: Targeted Modification of Gene Function Exploiting Homology-Directed Repair of TALEN-Mediated Double-Strand Breaks in Barley
Source: G3 (Bethesda). 2015 Jul 6;5(9):1857–63. doi: 10.1534/g3.115.018762 (PMC4555222; doi:10.1534/g3.115.018762)
Supplement: Supporting Information [file supp_g3.115.018762_FigureS2.pdf]

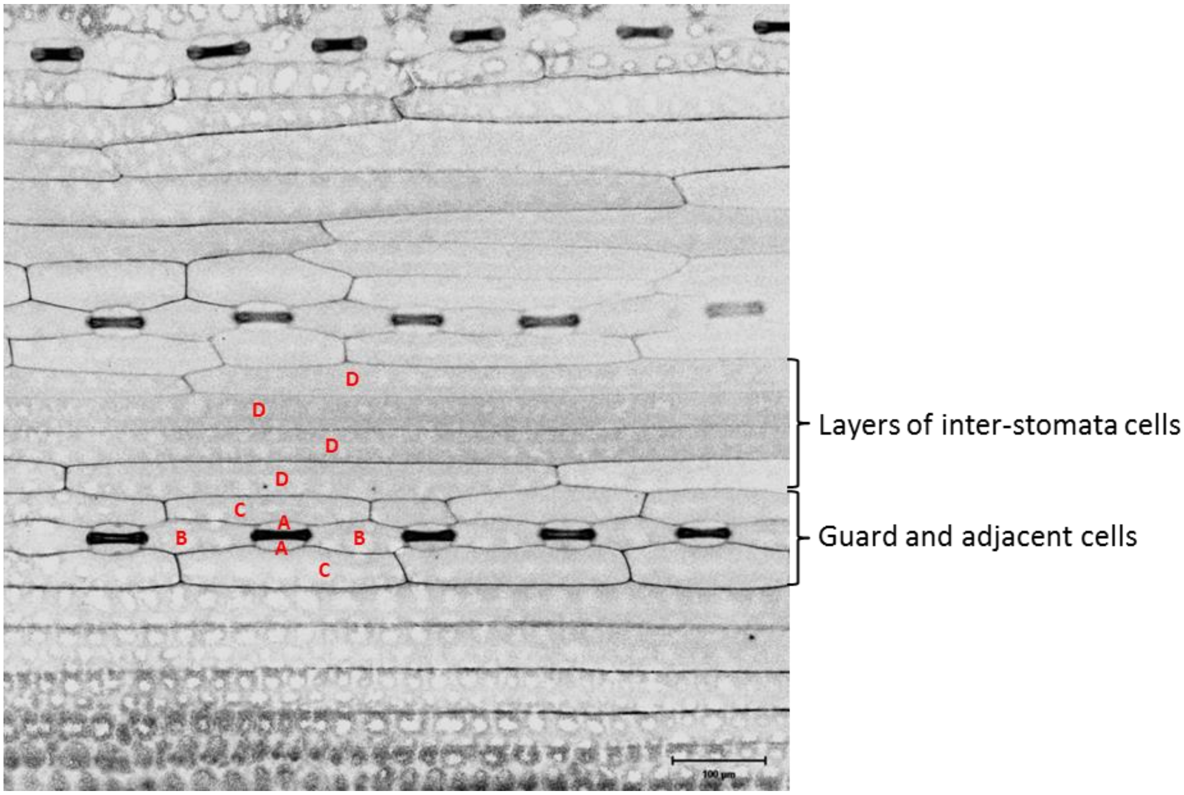

Figure S2. Confocal microscopy image of the barley abaxial leaf surface demonstrating the cell types present. The guard and adjacent epidermal cells labeled “A”, “B” and “C” were included in the count, whereas those marked “D” (narrow cells in the inter-stomatal region) were excluded due to the low efficiency of transient transgene expression.
