# Supplementary material for: Targeted Modification of Gene Function Exploiting Homology-Directed Repair of TALEN-Mediated Double-Strand Breaks in Barley
Source: G3 (Bethesda). 2015 Jul 6;5(9):1857–63. doi: 10.1534/g3.115.018762 (PMC4555222; doi:10.1534/g3.115.018762)
Supplement: Supporting Information [file supp_g3.115.018762_FigureS3.pdf]

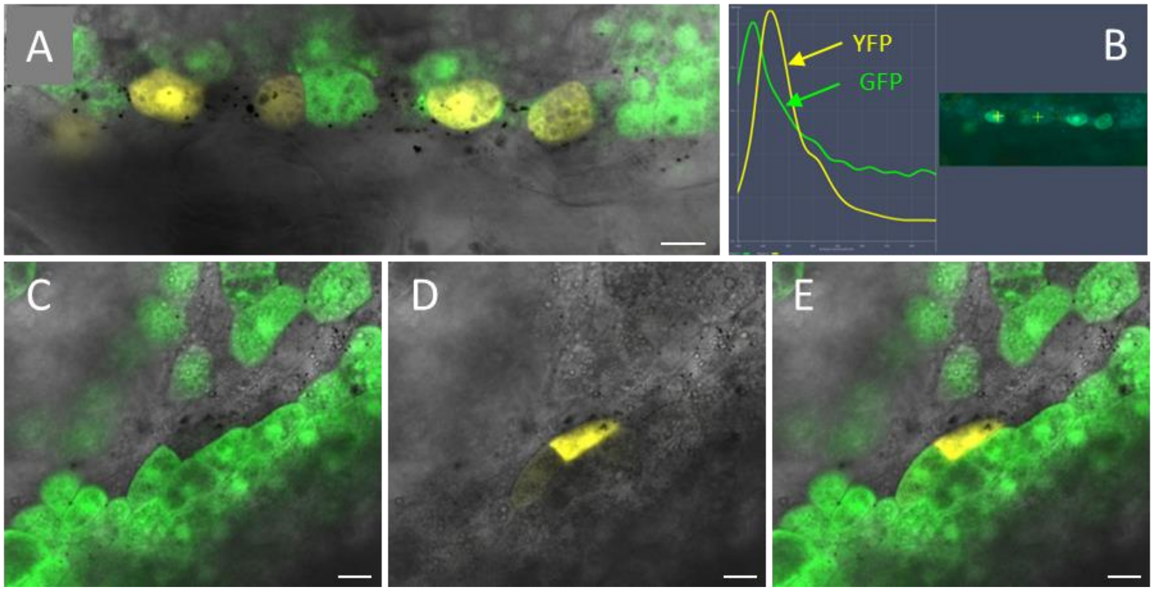

Figure S3. HDR following the induction of TALEN-mediated DSBs in cultured immature barley embryos. (A) Merged bright field and epifluorescence images of line 462L (carrying *gfp* and the left-hand TALEN unit) callus taken 24 h after bombardment with the right-hand *gfp*-TALEN unit and linearized *yfp** fragment. Bar: 20 µm. (B) Lambda stack of same materials shown in (A) used to visualize the presence of GFP (emission peak at 509 nm) and YFP (527 nm). (C; D) Epifluorescence of transiently transformed 462L callus after excitation with 488 nm laser light and spectral unmixing to identify (C) GFP and (D) YFP signals. Bar: 20 µm. (E) Merged image of (C) and (D).
