# Supplementary material for: Targeted Modification of Gene Function Exploiting Homology-Directed Repair of TALEN-Mediated Double-Strand Breaks in Barley
Source: G3 (Bethesda). 2015 Jul 6;5(9):1857–63. doi: 10.1534/g3.115.018762 (PMC4555222; doi:10.1534/g3.115.018762)
Supplement: Supporting Information [file supp_g3.115.018762_TableS1.pdf]

Table S1. List of primers used for the identification of T-DNA elements in the study.

Primer	Sequence 5' – 3'	Amplified region, primer orientation
Actin-F1	GGATCCGATGGCTGACGGTGAGGACATCCAG	*HvACTIN1*, forward
Actin-F2	CCATGGAGAAGCACTTCCTGTGGACGATCG	*HvACTN1*, reverse
FokI-F1	ATCGAGATCGCCCGGAACAGCACC	*Fok*I gene, forward
FokI-R	ATCATCTCGCCGCCGATCAGGAGC	*Fok*I gene, reverse
GH-35S-R1	GAGGCATCTTGAACGATAGC	*CaMV 35S* promoter, reverse
GH-YFP-TALEN-F2	CGACTTTAAAGAAGATGGTA	*yfp* TALEN-right unit, forward
